# Automatic Code Review by Learning the Structure Information of Code Graph

**DOI:** 10.3390/s23052551

**Published:** 2023-02-24

**Authors:** Ying Yin, Yuhai Zhao, Yiming Sun, Chen Chen

**Affiliations:** School of Computer Science and Engineering, Northeastern University, Shenyang 110169, China

**Keywords:** code review, program dependency graph, deep learning, CodeBERT

## Abstract

At present, the explosive growth of software code volume and quantity makes the code review process very labor-intensive and time-consuming. An automated code review model can assist in improving the efficiency of the process. Tufano et al., designed two automated tasks to help improve the efficiency of code review based on the deep learning approach, from two different perspectives, namely, the developer submitting the code and the code reviewer. However, they only used code sequence information and did not explore the logical structure information with a richer meaning of the code. To improve the learning of code structure information, a program dependency graph serialization algorithm PDG2Seq algorithm is proposed, which converts the program dependency graph into a unique graph code sequence in a lossless manner, while retaining the program structure information and semantic information. We then designed an automated code review model based on the pre-trained model CodeBERT architecture, which strengthens the learning of code information by fusing program structure information and code sequence information, and then fine-tuned the model according to the code review activity scene to complete the automatic modification of the code. To verify the efficiency of the algorithm, the two tasks in the experiment were compared with the best Algorithm 1-encoder/2-encoder. The experimental results show that the model we proposed has a significant improvement under the BLEU, Lewinshtein distance and ROUGE-L metrics.

## 1. Introduction

Code review is one of the essential activities in software development and maintenance. Code review refers to the systematic inspection of source code in the software life cycle. Its purpose is to find software defects or code irregularities, ensure the overall quality of the software, and enhance the stability of the system. However, the size of the current software system is gradually increasing, the function is more and more complex, and the problems existing in the software are also gradually increasing. According to the statistics of Google’s code review system [1], on average, every working day, about 20,000 code changes that meet the requirements are submitted. If the code review is not carried out on time and the repository of code not updated on time, the accumulation of problems will be more and more, and the security risks of the system will be more and more. At the same time, a variety of open-source software and open-source code review systems continue to emerge. The massive code, code changes, and interactive comments between persons in the system provide massive data support for code review research [2,3,4,5]. Therefore, we can learn from the massive data with the help of artificial intelligence, data mining, and other technologies to improve the efficiency of code review.

**Figure 1:**
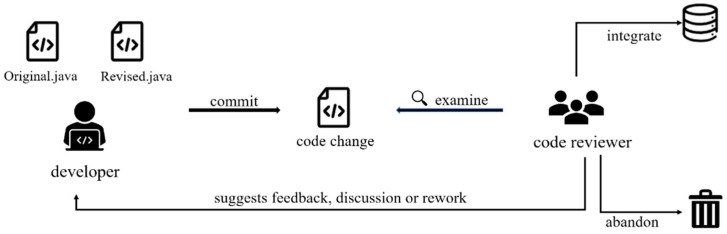
The process of code review.

Figure 1 shows the widely used lightweight code review process. Developers modify the code to solve the problems existing in the current software system, and then submit the code changes to the code review system, and the code review staff browses the relevant diff file. The file of diff describes the differences between the unmodified program file and modified program file in detail. The reviewer makes comments according to the problems in the code changes, and the developer remodifies the code according to the feedback. The whole process, such as commit, suggestion, feedback, rework, iteration, code reviewer, and patch submitter, is spread out by an online discussion system until both parties are satisfied. The last discussion about code change can be integrated into the code repository or abandoned. The primary purpose of code review is to find software defects. During the review process, developers can improve their ability by reading the source code, and analyzing and discussing the problems. Additionally, through the code review, the team can develop a good knowledge-sharing system, and enhance team awareness.

At present, many tools can help improve the efficiency of code review. The process of manual code review not only involves substantial human resources but also consumes a lot of time. From the perspective of developers, they need to find possible problems in the software system by carefully checking the source code. From the perspective of reviewers, they need to understand the purpose and history of the code intention, carefully read, understand, analyze and discuss the code change file submitted, make a fair evaluation of the code changes, and then provide feedback on the review comments to the developers. The above process is very heavy and time-consuming work. There is some work endeavoring to improve the efficiency of code review, such as automatically learning the features in the data through deep learning models [6,7,8,9,10]. These works propose a new design idea for automatic code reviews, such as using naive Transformer models and related datasets.

However, most of them do not sufficiently exploit the information contained in the code. While the code contains rich logical relationship and semantic information, such as data dependencies between codes, control dependencies, and the execution logic of the code, the effectiveness of the code review algorithm can be further improved by fully fusing the structural features of the graph. Therefore, this paper aims to improve the efficiency of code review by fusing program structure information and code sequence information to enhance the learning code information. This is one of the challenges of current code review.

To address this challenge, this paper enhances the code features learning from a different perspective by fusing program graph structure information with code sequence information, thus providing richer information on the decoder side. The contributions of this paper are as follows: (1) We propose an algorithm called PDG2Seq to serialize the program dependency graph, which transforms the program dependency graph into a unique graph sequence encoding in a lossless way. The graph sequence encoding not only contains the logical structure information of the program, but also preserves the semantic information of the nodes and edges of the program dependence graph; (2) We design an automatic code modification transformation model called crBERT, based on the pre-trained model CodeBERT, to combine the program semantics and structural features to enhance the learning ability of the code, and then fine-tune the parameters according to the code review task; (3) We carried out many comparative experiments on public datasets. In terms of BLEU, ROUGE-L, and Levenshtein distance metrics, the proposed method has a significant improvement compared with the current state-of-the-art methods in both tasks.

The structure of the paper is as follows. Section 1 is the introduction. Section 2 is the related works. Section 3 of this paper discusses the background. Section 4 elaborates on the problem statement and crBERT Model. Section 5 presents the experimental results. Section 6 is the summarization.

## 2. Related Works

### 2.1. Code Feature Representation

Code is written in a programming language that contains more information than natural language. How to extract the more abundant source code features more effectively has become a problem that has attracted more and more researchers. At present, there are roughly three ways to represent code features: natural language processing is used to extract the semantic information of code, abstract syntax tree is used to extract the syntactic features of code, and graph structure is used to extract the structural information of code. Sequence features of code and structural features of code have also become important features for code analysis in recent years.

In terms of extracting code sequence features, Xuan Huo et al. solved the software defect localization by exploring the sequential relationship of code statements [7]. This work exploits the structural and sequential nature of control flow graphs to improve feature identification for bug location.

Furthermore, a deep learning model CG-CNN is proposed to learn unified features for defect location.

After the birth of the BERT model [11], its outstanding performance has triggered a research boom in industry and academia, and there have been numerous variants of the pre-training model BERT used in different domains and for different tasks. Code BERT [10] is a general code representation model proposed by Feng et al. for the software engineering domain, which is a bimodal pre-training model based on programming language and the natural language model, which is based on both Masked Language Modeling (MLM) and Replaced Token Detection (RTD) tasks for pre-training. The MLM task uses a bimodal form of the natural and procedural language dataset by randomly masking out 15% of the words at random and then training the model to predict the token based on the context of that token. The RTD task is first trained separately on natural language and code language data to obtain a data generator to generate reasonable replacement words for the random mask positions, and a discriminator to detect whether a word is the original word to extract code sequence information.

In terms of extracting structural features of code, Wang et al. [12] argued that neural networks based on abstract syntax trees could learn structural features of programs, but these models could not capture different types of substructures in programs. Therefore, a modular tree network was proposed, which dynamically combines different neural network units into tree structures based on input abstract syntax trees. The network can capture semantic differences between different substructures. The DeepCom model [13] proposes a sequence-based language model to analyze the abstract syntax tree of a program. In order to better represent the syntactic structure of the abstract syntax tree and embedded semantic information, this work proposes a structure-based traversal (SBT) method to convert the abstract syntax tree into a sequence in as lossless a way as possible, and then use the sequence model to extract features from the SBT sequence. Wu et al. [14] proposed a structure-induced self-attentiveness mechanism that uses multi-view structural cues to encode sequential encoding inputs, where they represent the program in the form of an AST, on which they construct a multi-view graph based on syntax, data dependencies, and control dependencies, and then use the structure-induced Transformer to extract the code features. Alexander et al. [15] represented the program as abstract syntax trees and then used graph convolutional neural networks to learn the relationships between nodes in the trees to capture program graph features.

### 2.2. Code Review

In the field of software engineering, the research of automated code review is still in its initial stage. More and more researchers try to use artificial intelligence methods to solve various problems in code review, to improve the efficiency of the code review process. The current research is mainly based on traditional machine learning and deep learning.

The traditional machine learning-based method mainly manually designs features for datasets in the field of code review. Fan et al. [16] designed 34 features from five perspectives: code, file history, committer experience, cooperation network, and text, and uses the random forest to determine whether code changes can be committed to the code repository. Anderson et al. [17] also started from the above five perspectives to design 41 features to predict which code changes would have a significant impact on follow-up code review activities. They used six different machine learning algorithms, such as support vector machine and random forest to carry out the research. Soares et al. [18] manually designed 36 features using different classifiers to determine whether the code reviewers are suitable; they found that the numbers of committed code and social relationship between the code submitter and code reviewers have an impact on the recommendation of suitable code reviewers.

Deep learning models can automatically learn data features. Shi et al. proposed an algorithm to automatically determine whether a code change passes in code review [19]. In this work, the code changes are regarded as a binary classification task, and the features of code are extracted by using a convolutional neural network and LSTM network, and the differential features of code changes are extracted by using the paired autoencoder model. Heng-Yi Li et al. proposed a multi-instance-based automatic code review framework [20]. In this work, each code review is regarded as a multi-instance package containing multiple code change blocks, and an end-to-end model is proposed to extract the different features between the original code package and modified code package to predict whether the code review is passed. Siow et al. proposed a code review opinions recommendation method based on a given code change [21]; they use a deep learning model and an attention mechanism to perform character-level and word-level multiple semantic embeddings to extract multivariate semantic features. Thong et al. proposed CC2Vec [22], a deep learning model, which models the hierarchy of code changes with the help of an attention mechanism and uses multiple comparison functions to identify the differences between before and after code changes. Xin Ye et al. proposed a multi-instance-based deep learning model [23]; the model recommends appropriate code reviewers by learning code changes, commit information, titles, and so on. Yao Wan et al. [8] proposed to use deep reinforcement learning to automate code summary generation. They used the LSTM model to learn the semantic features of the code; the tree recursive neural network (tree-RNN) model was used to learn the abstract syntax tree features of the code, and the two features were fused as the final code features, and then the code features were re-adjusted by the deep reinforcement learning evaluation. However, this work only uses the semantic and syntactic features of the code, ignoring the structural features of the code. In summary, most of the above studies on code review only use raw code sequences as input and do not explore code features in depth. Code sequences can not only provide semantic information for the model, but also have rich structural information hidden in the program, such as data dependency between codes, control dependency, and code execution logic. However, most of the related works on code review only use the original code sequence as input and do not deeply explore the code characteristics. However, code sequences not only provide semantic information, but also potentially contain rich structural information, such as data dependencies, control dependencies, and execution logic between codes.

To fully capture the structural features of the code, we transform the source code into a program dependency diagram at the level of method granularity, which provides a good representation of the control flow, data dependencies, and control dependencies of the code. Based on the code sequence information, the program diagram structure information is integrated to strengthen the model’s learning of code features from different perspectives, thus providing richer information on the decoder side. CodeBERT pre-training uses code data and code-related natural language descriptions for training. The model can learn prior knowledge about code and natural language from massive pre-training data. The data in this paper includes not only code data, but also comments from reviewers. Therefore, this paper uses CodeBERT to extract deeper code features and comment features, and then fine-tune the parameters according to actual code review task scenarios.

To make full use of the code sequence features and structural information in the program dependence graph, we use graph sequence encoding to learn the program dependence graph features.

## 3. Background

### 3.1. Program Dependence Graph (PDG)

The source files are written in a programming language that is close to the natural language. The code contains sufficient semantic information, such as “int” stands for integer variables, and the identifier “printEmployeeSalary” can be broken down to represent the semantics of printing employee’’ salaries. Therefore, the use of natural language processing technology can effectively extract the semantic features of the program. However, code is different from natural language. The source code contains very rich and complex logical structure features. For example, in the switch structure, the program will selectively execute the corresponding code block according to different judgment conditions, rather than executing all the statements in order. For another example, a variable defined in the previous may be referenced many times later, and there might be some strong dependencies in the data. Therefore, it becomes particularly important to learn the underlying logical structure information in the feature representation of the code.

In the field of software engineering, graph models such as the control flow graph (CFG), data dependence graph (DDG), and program dependence graph (PDG) are commonly used to represent the structural information of programs. Different models emphasize different representations of information. The control flow chart shows the possible flow of all the basic blocks in a process. A data dependence graph is used to represent the dependencies among the data in a statement block. A program dependence graph is a synthesis of a control flow graph and data dependence graph, which contains not only a control dependence relation but also dependencies between variables. Therefore, we use a program dependence graph to represent the structural characteristics of programs. 

**Figure 2:**
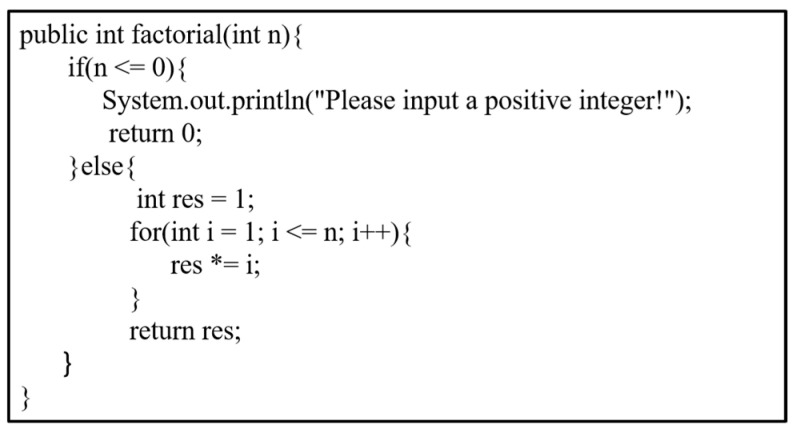
Factorial function of the positive integer *n*.

**Figure 3:**
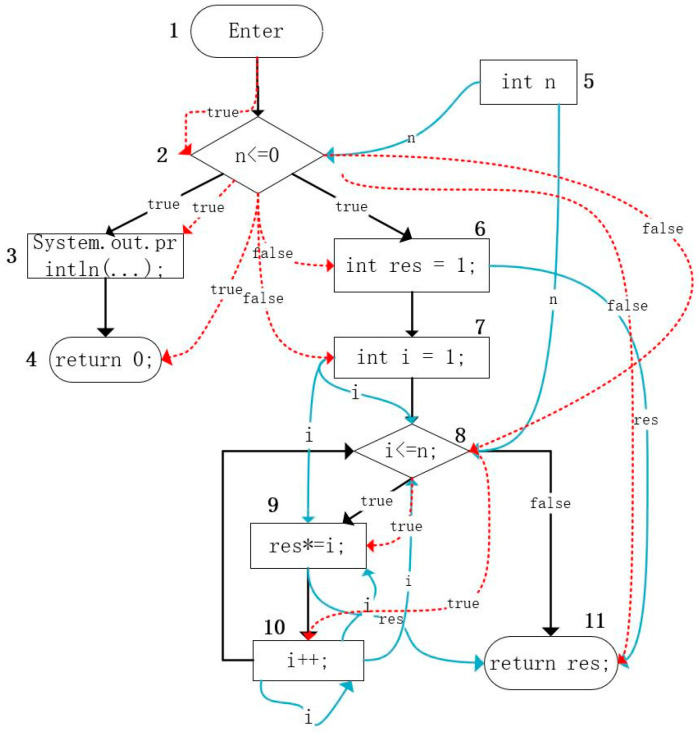
Program dependency graph.

Figure 2 shows a function to calculate the factorial of a positive integer n. Figure 3 shows the corresponding program dependence graph of Figure 2. A complete program dependence graph includes the beginning node, execution nodes, and ending node. The rectangle with rounded corners represents the beginning node or the ending node, the diamond represents the condition of judgment, and the rectangle represents the execution process. The program dependency graph contains not only the structural features of the code but also the semantic information of the program. Each node corresponds to the corresponding code block statement of the program. A program dependence graph is the fusion of a control flow graph, control dependence graph, and data dependence graph. For detailed information, the black figures and arrows represent the control flow graph of the program, and the black directed edges in the graph represent the logical execution order of the two codes. The blue arrow represents the data dependence relationship in the code, and the identifier on the arrow represents the relevant variable of the data dependence. The data dependency represents the transfer and flow of data between data variables in the program. For example, the formal parameter “n” is dependent on the data in the two judgment statements (the two diamonds) in Figure 3. The red arrow in Figure 3 represents the potential control dependence of the program, such as if node 2 is not executed, the subsequent nodes 3, 4, 6, 7, 8, and 11 cannot be executed, and if and only if the judgment condition of node 2 is true, the subsequent nodes 3, 4 can be executed. Similarly, only if the judgment condition of node 2 is false, the subsequence nodes 6, 7, 8, and 11 can be executed.

### 3.2. Minimum DFS Encoding

When using depth-first traversal on a graph, choosing different initial nodes and intermediate nodes results in different depth-first search sequences. In the frequent subgraph mining algorithm gSpan, the lexicographical order-based minimal DFS encoding, which is a formal description of graphs, was proposed [24]. Li et al. [25] proved that the minimum DFS encoding is a serialization code corresponding to the graph. The serialization code can convert the traversed graph into the form of a sequence, which greatly reduces the time and space complexity of the algorithm. Inspired by gSpan, this paper proposes a program dependence graph serialization algorithm, PDG2Seq, which can preserve the structural and semantic information of the program only by using the minimal program dependence graph sequence encoding and effectively avoid the computational overhead by repeating candidate sets caused by different traversal orders. Next, we introduced the basic concepts involved in the graph serialization process.

A labeled graph is a representation of a graph structure, denoted as G = (V, E, L(V), *I*), *L*), where *V* = {*v*_1_, *v*_2_,…, *v_n_*} is the nodes set of the graph, *E* = {*e_ij_* = (*v_i_*, *v_j_*)| *v_i_*, *v_j_*∈*V*} represents the set of edges of the graph, and the function L represents the label mapping function, which maps the nodes and edges in the graph structure to their corresponding labels.

**Figure 4:**
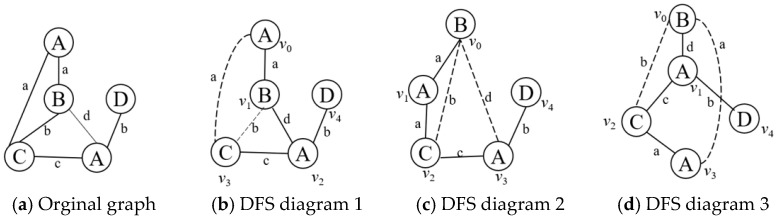
Depth-first search subgraph.

Different traversal methods will produce several different depth-first sequences. Figure 4b–d are different depth-first search subgraphs corresponding to Figure 4a. The solid-line connected parts of the diagrams are the corresponding depth-first search (DFS) trees. *v_i_* indicates the order in which the nodes are accessed during the depth-first search, with upper-case letters indicating the n’des’ labels and lower-case letters indicating the e’ges’ labels. The edges belonging to the DFS tree in the figure are the forward edges, i.e., the solid parts of Figure 4b–d; the edges not belonging to the DFS tree in the figure are the backward edges, i.e., the dashed parts of Figure 4b–d; let the edge *e* = (*v_i_*, *v_j_*), then the forward edges satisfy *i* < *j* and the backward edges satisfy *i* > *j*.

Define the linear order rules: Assume *e*_1_ = (*i*_1_, *j*_1_), *e*_2_ = (*i*_2_, *j*_2_), if there exists *i*_1_ = *i*_2_ and *j*_1_ < *j*_2_, then $e_{1}{≺}_{T}e_{2}$; if $e_{1}{≺}_{T}e_{2}$ and $e_{2}{≺}_{T}e_{3}$, then $e_{1}{≺}_{T}e_{3}$. DFS encoding is a depth-first search of the graph according to the ${≺}_{T}$ rule that expands the subgraph from an edge. Finally, the corresponding sequence of edges $\left(e_{1},e_{2},\ldots ,e_{n}\right)$ is obtained. The sequence of edges satisfies $e_{i}{≺}_{T}e_{i+1}$, and each edge will contain five elements, which represent the start point, end point, label of the start point, label of the edge, and label of the end point. For example, the edge $\left(v_{0},v_{1}\right)$ in Figure 4b is denoted as $\left(v_{0},v_{1},A,a,B\right)$. Table 1 shows the depth-first coding sequences generated according to the rules ${≺}_{T}$ in Figure 4b–d.

The DFS dictionary order represents the size relationship between sequences of edges. Since there may be multiple encoding sequences for a graph, we can obtain the minimum DFS encoding of the graph by minimizing the dictionary order. Let Z be the set of all depth-first search sequences of graph G. Let $\alpha =\left(a_{0},a_{1},\ldots ,a_{m}\right)$, $\beta =\left(b_{0},b_{1},\ldots ,b_{n}\right)$, where α,β∈Z. The set of forward edges and backward edges corresponding to α,β are $E_{\alpha ,f},E_{\alpha ,b},E_{\beta ,f},E_{\beta ,b}$ respectively, where, $\alpha_{t}=\left(i_{a},j_{a},l_{i_{a}},l_{\left(i_{a},j_{a}\right)},l_{j_{a}}\right)$, $b_{t}=\left(i_{b},j_{b},l_{i_{b}},l_{\left(i_{b},j_{b}\right)},l_{j_{b}}\right)$. If α,β satisfies the condition: (1) or (2) in Equation (1) below, then α≤β.

For example, the first edge subscript of both column (c) and column (d) of Table 1 is (0,1), and the markings of both the forward edge and starting node are B. Therefore, according to rule 5 in condition (1), the marking dictionary order sizes of the two edges are compared, i.e., a<d, so $\left(v_{0},v_{1},B,a,A\right){≺}_{T}\left(v_{0},v_{1},B,d,A\right)$, that sequence (c) <sequence (d).

(1)
$\exists t,0\leq t\leq \min\{m,n\},a_{k}=b_{k},\mathrm{when}\;k<t\;\mathrm{and}$
(1) $$a_{t}<b_{t}=\{\begin{array}{c} \mathrm{true}\;\mathrm{if}\;a_{t}\in E_{\alpha ,b}\;\mathrm{and}\;b_{t}\in E_{\beta ,f}.\\ \mathrm{true}\;\mathrm{if}\;a_{t}\in E_{\alpha ,b},b_{t}\in E_{\beta ,b},and\;j_{a}\;<\;j_{b}.\\ \mathrm{true}\;\mathrm{if}\;a_{t}\in E_{\alpha ,b},b_{t}\in E_{\beta ,b},and\;j_{a}=j_{b},and\;l_{\left(i_{a},j_{a}\right)}<l_{\left(i_{b},j_{b}\right)}.\\ \mathrm{true}\;\mathrm{if}\;a_{t}\in E_{\alpha ,f},b_{t}\in E_{\beta ,f},and\;i_{b}\;<\;i_{a}.\\ \mathrm{true}\;\mathrm{if}\;a_{t}\in E_{\alpha ,f},b_{t}\in E_{\beta ,f},and\;i_{a}=i_{b},and\;l_{i_{a}}<\;l_{i_{b}}.\\ \mathrm{true}\;\mathrm{if}\;a_{t}\in E_{\alpha ,f},b_{t}\in E_{\beta ,f},and\;i_{a}=i_{b},l_{i_{a}}=l_{i_{b}},and\;l_{\left(i_{a},j_{a}\right)}<l_{\left(i_{b},j_{b}\right)}.\\ \mathrm{true}\;\mathrm{if}\;a_{t}\in E_{\alpha ,f},b_{t}\in E_{\beta ,f},and\;i_{a}=i_{b},l_{i_{a}}=l_{i_{b}},l_{\left(i_{a},j_{a}\right)}=l_{\left(i_{b},j_{b}\right)},and\;l_{j_{a}}<\;l_{j_{b}}. \end{array}$$
(2)
$a_{k}=b_{k},\;\mathrm{when}\;0\leq k\leq m,\;\mathrm{and}\;n\geq m.$

## 4. The Algorithm Model

### 4.1. Problem Description

**Figure 5:**
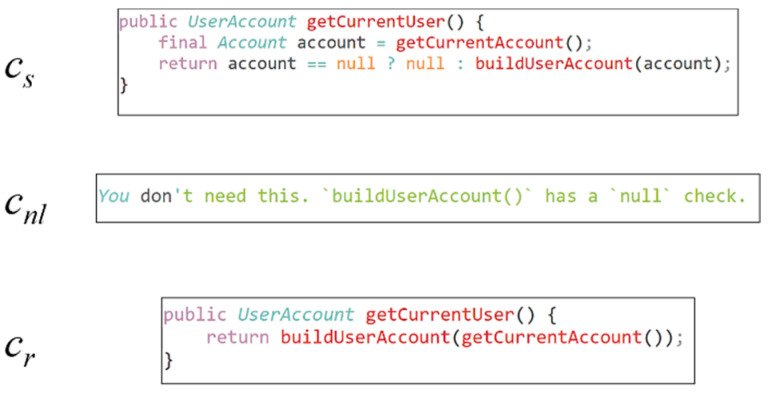
A sample in a dataset.

Code review activities involve multiple modified source files and functions. In practice, the granularity of the objects is diverse and very complex. This paper uses a lightweight code review approach based on code changes, i.e., code review at the granularity of methods. Figure 5 is a set of samples; *c_s_* = (*s*_1_, *s*_2_, …, *s_m_*) is the sequence of code generated by the developer after making changes to the code. After the code is submitted to the system, the reviewer makes a suggestion if *c_s_* iI *c_nl_* = (*n*_1_, *n*_2_, …, *n_p_*). The developer will generate a modified code *c_r_* = (*r*_1_, *r*_2_, …, *r_n_*) based on the suggestions *c_nl_*. *c_gs_* = (*gs*_1_, *gs*_2_, …, *gs_q_*) represents the coding sequence corresponding to the program dependency graph of the code sequence *c_s_*. The coding sequence *c_gs_* is obtained by the PDG2Seq algorithm proposed in this paper.

**Figure 6:**
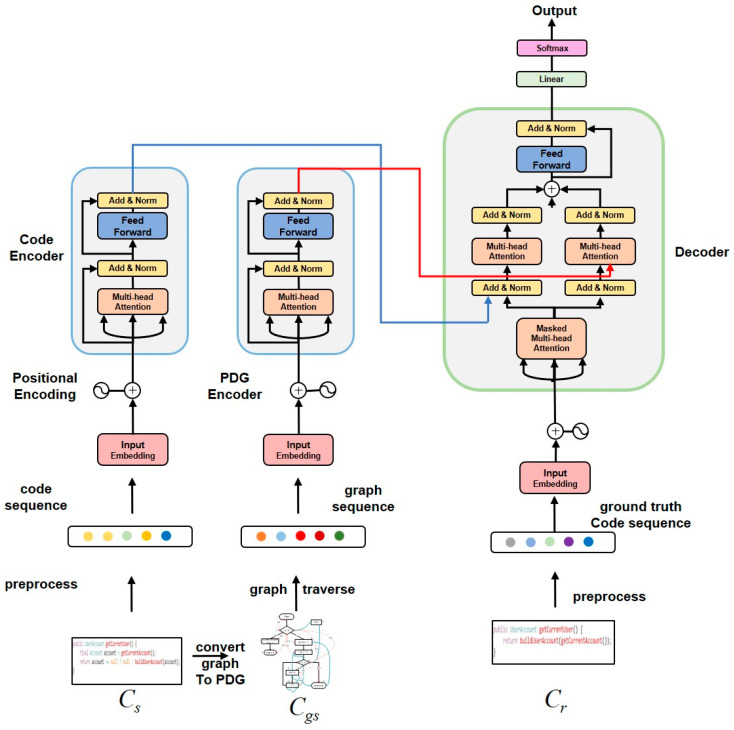
Task 1 corresponds to the model framework crBERT1.

Figure 6 shows the algorithmic framework of the corresponding model crBERT1 for Task 1. The crBERT1 model consists of three components: the code sequence feature extractor (CodeEncoder), the program graph sequence feature extractor (GSEncoder), and Decoder. The input of the crBERT1 model consists of original code sequences *c_s_’*, program dependent graph sequences *c_gs_’*, and the modified code sequence *c_r_*.

This paper generated the sequences *c_gs_* by fusing the PDG2Seq proposed in this paper based on the original code sequences features; the sequences *c_gs_* contain the graph structure information and semantic information of the program-dependent graph. The process can efficiently enhance the model’s learning of the code logic structure information. The problem is defined as follows: we first convert each method to the corresponding program dependency graph (PDG) and then generate the unique graph sequence corresponding to that PDF according to the PDG2Seq. The model crBERT1 learns a mapping from (*c_s_’c_gs_*) to *c_r_* with the dataset (*c_s_’c_gs_’c_r_*), by maximizing the conditional probability $\hat{c_{r}}=argmax_{c_{r}}P(c_{r}|c_{s},c_{gs})’$ to produce a code sequence $\hat{c_{r}}=\left({\hat{r}}_{1},{\hat{r}}_{2},\ldots ,{\hat{r}}_{n}\right)$ that is closest to that suggested by the code reviewer.

**Figure 7:**
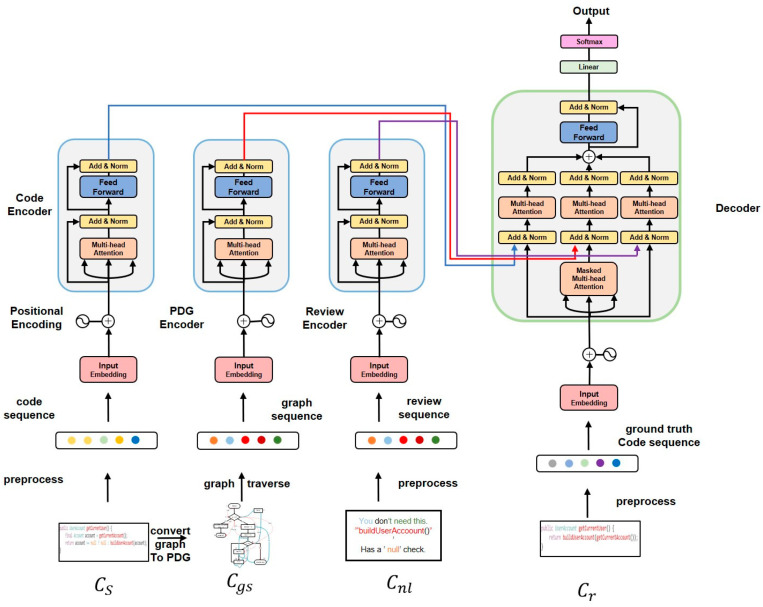
Task 2 corresponds to the model framework crBERT2.

Figure 7 shows the model crBERT2 corresponding to task 2. The crBERT2 model consists of four parts: the code sequence feature extractor (CodeEncoder), program graph sequence feature extractor (GSEncoder), review sequence feature extractor (ReviewEncoder), and decoder. On the decoder side, the modified code is decoded and output by fusing the code sequence features, code graph sequence features, and review sequence features.

For example, in this task, a developer submits code *c_s_* to the code review system, the reviewer makes a suggestion *c_nl_* according to *c_s_* and the crBERT2 model will automatically modify the code *c_s_* to code *c_r_* based on the suggestion *c_nl_*. In this way, when the review system gives feedback to developers, it can not only give feedback on suggestions described in natural language but can also attach that suggested code, giving developers a more intuitive understanding of the reviewer’s intentions and improving development efficiency. Therefore, the problem definition for Task 2 is: Model crBERT2 learns the mapping from < *c_s_’c_gs_c_nl_* > to *c_r_* with dataset < *c_s_’c_gs_’c_nl_c_r_’* > by maximizing the conditional probability $\hat{c_{r}}=argmax_{c_{r}}P(c_{r}|c_{s},c_{gs},c_{nl})\text{′}$, producing a code sequence that is closest to the description suggested by the code reviewer.

### 4.2. PDG2Seq Algorithm

The structure of a program graph contains very rich information. Using a program dependency graph to represent code not only captures structural information such as control dependencies and data dependencies of the program but also enhances the learning of program semantic information. This requires the crBERT model to be able to effectively extract features of the program dependency graph. Some techniques such as graph embedding and graph neural networks are current research hotspots in graph representation learning. However, these techniques are not suitable for extracting features of the program-dependent graphs for this paper, because the number of nodes in all graphs in the current dataset is less than 20, and this situation will result in the problem that the node feature information in the graph tends to be consistent, making the graph neural network too smooth [26]. Therefore, to be able to effectively learn the graph structure information of a program while retaining the semantic information in the program-dependent graph, this paper proposes a program-dependent graph serialization algorithm PDG2Seq inspired by minimal DFS encoding to generate a unique serialized representation of the graph with specified encoding rules.

Algorithm1 shows the detailed procedure of the PDG2Seq algorithm. In step 1 of the algorithm, we first specify that the entry node (Enter node) of the program dependency graph is the entry node of the graph sequence extension. Steps 2–4 initialize the access order of nodes. In step 5, the function DFSSearch in steps 10 to step 26 constructs a DFS-spanning tree illuminated by the idea of a greedy algorithm. In this case, when traversing each node, the edge with the smallest dictionary order is first selected for expansion, and that edge is added to the sequence. If a node has both control-dependent and data-dependent edges, the control-dependent edge is considered to have higher priority and the control-dependent edge is visited first; if a node has only multiple data-dependent edges or only multiple control-dependent edges, then the edge corresponding to the minimum lexicographic order is visited first. After the DFSSearch function, the minimum forward edges sequence *seq* and the set of backtracking edges E are obtained. In steps 6–7, according to the edge rules ${≺}_{T}$ and lexicographic order, the algorithm will insert the backtracking edges into the sequence *seq*. The insertion of backtracking edges satisfies the following rules: (1) For a given vertex *v*, its forward edge, (*v_i_*, *v_j_*) satisfies *i* < *j*, its backward edge (*v_i_*, *v_k_*) satisfies *i* > *k*, so the backward edge of *v* should appear in front of its forward edge; (2) If node v has more than one backtracking edge, it is inserted after sorting by lexicographic order; (3) If the vertex v has no forward edge, insert its backward edge after the edge with v as the terminal node. In this way, during the DFS traversal, the forward edge sequence we get is the smallest. According to the extension rule of the backtracking edge, the sequence is still the smallest in lexicographic order after adding the backtracking edge.
**Algorithm 1** Program dependency graph serialization algorithm PDG2Seq Input: G=(V,E), $V=\left\{v_{1},v_{2},\ldots ,v_{n}\right\}$, $E=\left\{e_{1},e_{2},\ldots ,e_{m}\right\}$ Output: **Minimum graph serialization encoding** $seq=\left\{s_{1},s_{2},\ldots ,s_{w}\right\}$
**1: **initialization seq**= (), i = 0; currentNode = *v*_0_;** **2: **for **v in V** do **3:   v.visted = −1;** **4: **end for **5: DFSSearch (G, currentNode,**
seq); **6: **for **e in E** do **7:  **Find the insertion position in *seq* according to the rule ${≺}_{T}$**,**Insert the backtracking edge e into *seq*; **8: **end for **9: **return seq **10: **Subprocedure 1 **DFSSearch (G, currentNode,** *seq*) **11:**   **currenINode.visited = i;** **12:   **for **nei in currentNode.neighbors** do **13:**    **array = ()** **14:**    for **e in E** do: **15:**      if **e.begin == currentNode** then **16:**        **array.add (e)** **17:**       **end if** **18:**    end for **19:    e = min (Sort (array));** **20:   ** if **q.visited == −1** then **21:**      **i + = 1;** **22:**      **E.remove (e);** **23:**      *seq***.add (e);** **24:**      **DFSearch (G,q,** *seq*); **25:    **end if **26:**   end for

**Figure 8:**
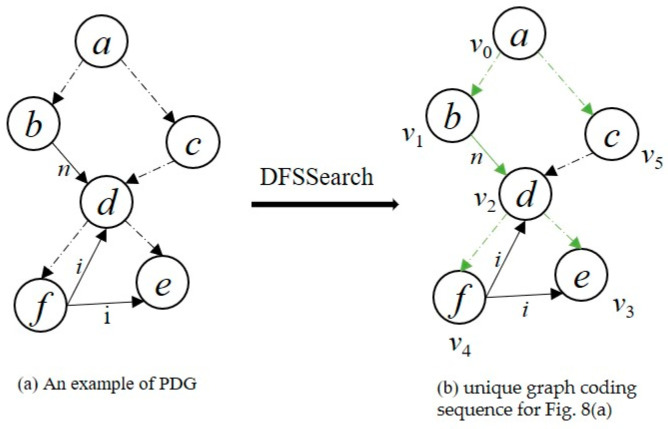
PDG2Seq algorithm example.

To more clearly describe how PDG2Seq converts the program dependency graph into the corresponding unique graph coding sequence, this paper takes the simplified program dependency graph in Figure 8a as an example to describe the process of generating the minimum graph coding sequence. In Figure 8a, the solid line represents the data dependency between nodes, and the marks at the edges of the solid line represent the variables of data dependency between nodes. The dotted line represents the potential control dependency relationship between nodes, and the mark on the edge of the dotted line represents the type of edge. In this paper, the mark on the control dependency edge is set to 0. Figure 8a,b show the process of generating a sequence of minimal forward edges of a graph. The green line in Figure 8b is the forward edge, and the black line is the backtracking edge. In the following, the process is described in detail with node a as an example: node a is the entry node of the program, and there are two control-dependent edges from node a. Since the dictionary sequence of edge (a,0,b) is smaller than the edge (a,0,c), node b is given priority access and (v0,v1,a,0,b) is added to the sequence. Then, the depth-first algorithm is used to traverse all nodes in the graph. After a depth-first search, the access order of nodes is determined to be v0 to v5 in Figure 8b according to the minimum lexicographical rule. After the current edge is extended, the traceback edge should be inserted into the graph encoding sequence according to the traceback edge extension rules. For example, node f in Figure 8b has two backtracking edges. According to rule (2), these two backtracking edges should be sorted first, that is, *p*(*v*_4_,*v*_2_,f,i,d)${≺}_{T}$*q*(*v*_4_,*v*_3_,f,i,e), and node f has no forward edge starting from this node. According to rule (3), the edges p and q should be placed after edge (*v*_2_,*v*_4_) in turn. The third column of Table 2 shows the unique minimum graph depth-first encoding sequence generated by the PDG2Seq algorithm. After removing some redundant information, the encoding sequence is converted to abbnddedffidfieaccd, and after removing the adjacent node attribute information that occurs multiple times in the generated sequence, the simplified graph encoding sequence is abndedfidfieacd.

### 4.3. crBERT Model

The CodeBERT pre-training task uses two different types of data, program language, and natural language to pre-train the model. Through the prior knowledge of code and natural language learning from massive data, it can capture the semantic features of code and the semantic features of reviewers’ comments on a deeper level. This paper uses the CodeBERT architecture to accomplish automated code review tasks. Figure 6 and Figure 7 are crBERT1 and crBERT2 models corresponding to the two tasks in this paper, where CodeEncoder is a code semantic feature extractor, PDGEncoder is a program diagram sequence feature extractor, and Review’ncoder is a reviewer’s comments feature extractor. *c_s_* and *c_nl_*, as input to the encoder after preprocessing and abstraction. For *c_gs_’* it is first converted into the corresponding program-dependent graph using existing tools, and then a unique sequence of graphs corresponding to that program is generated according to the PDG2Seq algorithm as input to the PDGEncoder.

Instead of using a recursive RNN structure, the Transformer uses global information, which makes it difficult for the Transformer to take advantage of highly important sequential information. However, the Transformer can use positional encoding to represent the positional information of words. Therefore, this paper adopts the positional encoding method proposed in the literature [27], which is calculated as shown in Equations (2) and (3), where pos denotes the position in the sequence, *d* denotes the dimension of the embedding, 2*i* denotes the even dimension and 2*i* + 1 denotes the odd dimension. The sum of the positional embedding of the word and the semantic embedding of the word will be used as the final vector of the word.

(2) $$PE\left(pos,2i\right)=\sin(pos/10000^{2i/d})$$

(3) $$PE\left(pos,2i+1\right)=\cos(pos/10000^{\left(2i+1\right)/d})$$

Each encoder is composed of several identical modules. Each module includes several parts: a multi-attention mechanism layer, residual layer, layer normalization, and linear transformation. The multi-head attention layer model adopts the idea of a self-attention mechanism to learn the sequential representation of the current word by calculating the relationship between the current word and other words at other positions in the sequence. Equation (4) is a calculation of self-attentiveness, in which *Q*, *K,* and *V* are obtained by multiplying the input or output of the previous layer by the corresponding parameter matrix $W^{Q},W^{K},W^{V}$, respectively. Q and each K calculate the intensity of attention between the two words by the dot product similarity, to prevent the inner product from being too large divided by the square root of dimension *d_k_*.

(4) $$Attention(Q,K,V)=softmax(\frac{QK^{T}}{\sqrt{d_{k}}})V$$

To obtain different context characteristics of multiple subspaces, the idea of multiple mechanisms is adopted in this paper.

The calculation process of the multi-head attention mechanism is shown in Equation (5), in which the output of each part is stitched and linearly transformed through multiple calculations of self-attention from different angles, and finally used as the final output of the current layer, where $head_{i}$ represents the output of the i-th head of self-attention, and *n* is the number of attention heads, $W^{O}$ is the weight matrix corresponding to the stitching of the *n* head attention outputs, $W_{i}^{Q},W_{i}^{K},W_{i}^{V}$ correspond to the weight matrix of Q,K,V respectively. The residual layer and normalization layer can effectively avoid the problem of gradient disappearance and weight matrix degradation by the residual connection and normalization.

(5) $$MultiHead(Q,K,V)=concat(head_{1},head_{2},\ldots ,head_{n})W^{O}\;head_{i}=Attention(QW_{i}^{Q},KW_{i}^{K},VW_{i}^{V})$$

The decoder and encoder have a similar structure. On the decoder side, the hidden layer states of different encoders are connected by concatenation operation, and then a linear transform and tanh non-linear transform are used to produce multiple features after fusion. Where $W_{c}$ represents the weight matrix and h represents the hidden layer state of each encoder. Equations (6) and (7) represent the feature fusion process of crBERT1 and crBERT2 model encoders, respectively. The output vector O of the final layer is transformed by softmax to obtain the corresponding output vector ${\hat{r}}_{i}$, as shown in Equation (8).

(6) $$h=\tan h(W_{c}\left[h_{s};h_{gs}\right])$$

(7) $$h=\tan h(W_{c}\left[h_{s};h_{gs};h_{nl}\right])$$

(8) $$Y_{\hat{r}i}=softmax(W^{y}O+b)$$

## 5. Experiment and Analysis

### 5.1. Description of Experimental Dataset

This paper uses an open-source dataset provided by Tufano et al. [6]. The dataset contains information on the pre-submission code, reviewer comments, and code modified based on comments crawled from the code review platforms Gerrit and GitHub, which corresponds to the dataset sample triplet <$c_{s}$,$c_{nl}$,$c_{r}$> as shown in Figure 5.

In a code study using the Seq2Seq model, the literature [28] found that code abstraction techniques can greatly reduce the size of the lexicon and improve model learning. Therefore, the author used the src2abs tool to realize code abstraction. For example, the first variable that appears in a method is replaced with VAR_1, the second variable that appears is replaced with VAR_2, the first method name that appears in that method is replaced with Method_1, and so on. The process takes into account all identifiers and does not abstract for some frequently used identifiers such as length (), List, etc. To ensure consistency, while the method is being abstracted, the corresponding comment is also mapped. For example, the comment “convert date to String” is also modified to “convert VAR_1 to String” due to the variable data being abstracted VAR_1 in the code. Additionally, the authors define some heuristic rules to filter out irrelevant comments, such as one-word comments (e.g., “thanks”), requests to change the format without affecting the code, and so on. In this paper, some samples that cannot be converted into program dependency graphs are filtered, and the final number of training, validation, and test set containing quaternions < $c_{s}$,$c_{gs}$,$c_{nl}$,$c_{r}$ > is 12,727, 1584, and 1598, respectively.

### 5.2. Evaluation Metrics

In this paper, BLEU, Lewenshtein distance, and ROUGE-L metrics in natural language processing are used to evaluate the effectiveness of the algorithm.

The BLEU [29] (bilingual evaluation understudy) metric was first used to evaluate machine translation. It calculates text similIrity by counting the n-gram cases of overlap between two given texts; n is the number of consecutive words.

Equations (9) and (10) are the calculation methods of BLEU, where $p_{n}$ represents the accuracy rate of n-gram of the generated sample and reference sample. This paper uses the average value of BLEU-1 to BLEU-4 as the final evaluation metric, corresponding to a weight $w_{n}$ = 0.25, where c is the length of the generated text and r is the length of the reference text.

(9) $$BLEU=Be^{\overset{4}{\underset{n=1}{\sum }}w_{n}\log(p_{n})}$$

(10) $$B=\{\begin{array}{cc} 1, & c>r\\ e^{1-r/c}, & c\leq r \end{array}$$

The Levenshtein distance is a metric used to evaluate the distance between two strings sequences, and it is a type of edit distance. The Levenshtein distance between two sequences is the minimum number of word edits (e.g., delete, insert, and substitute) required for one sequence to become the other. In this study, this edit distance is divided by the maximum length of the generated text and predicted text for normalization operations.

ROUGE [27] (recall-oriented understudy for gisting evaluation). This metric is similar to the BLEU metric in that the BLEU metric focuses on accuracy, while the ROUGE metric focuses on completeness. This paper uses the ROUGE-L indicator of the ROUGE metrics, which compares the similarity between texts by calculating the common subsequence between two texts.

In the testing phase, the BeamSearch strategy is used to generate multiple sequential solutions. The method considers not only the optimal solution that minimizes the loss function but also the k solutions with the highest conditional probability. For the first time step, k words with the highest probability are selected as the first word of k sequences. For each subsequent time step, based on the output sequence of the previous time step, k sequences with the highest probability in all combinations are selected as the current output sequence. Finally, k sequences with the highest conditional probability are generated. In this study, the search space of k = 1, 3, 5, and 10 are used, respectively, and the sequence with the highest BLEU score in k-generated sequences is selected as the optimal solution.

### 5.3. Experimental Parameter Settings

In this study, the dataset is randomly divided into a training set, verification set, and testing set. We use the best-performing model on the validation set as the final learning model. The length of code sequences, program diagram sequences, and review comment sequences in this study is not more than 100. When the length is less than 100, 0 is used to supplement the alignment, and when the length is more than 100, truncation is carried out. The Dropout mechanism was used to avoid overfitting, and the parameter was set to 0.5. The word vector and hidden dimension of the experiments are both 768 dimensions. Each encoder consists of 12 layers of identical modules, and the decoder is set to 6 layers. During training, the size of each data batch was set to 16; the Adam optimizer was used for gradient descent, the learning rate was set to 5e–5, and a 12-head attention mechanism was adopted.

### 5.4. Experimental Results and Analysis

The crBERT1/crBERT2 model proposed in this study uses the pre-trained model CodeBERT with prior knowledge to extract deeper sequence features, in addition to which we incorporate more information-rich program structure information based on semantic features. To explore the effectiveness of the crBERT1/crBERT2 model for tasks one and two, we compare it with the state-of-the-art 1-encoder/2-encoders model for solving this task. 1-encoder/2-encoders is the first automated code review model based on deep learning proposed by Tufano et al. [6]. While the model treats code as sequences, it does not explore the semantic features of deep code and structural features of code.

The experimental results of BLEU are shown in Table 3 and Table 4. Table 3 is the experimental results of the Task 1 BLEU metric, and Table 4 is the experimental results of the Task 2 BLEU metric. To demonstrate the effectiveness of the algorithm, we adopted the BeamSearch strategy to explore multiple solutions with beam sizes of 1, 3, 5, and 10, and selected the sequence with the highest BLEU score in the generated sequence as the optimal solution. It can be observed from the experimental results that the crBERT1/crBERT2 model has obvious improvement in task 1 and Task 2. In task 1, crBERT1 has been improved by 8.8, 6.1, 5.5, and 4.8%, respectively, under different BeamSize compared with the 1-encoder model. In task 2, crBERT2 has been improved by 3.6, 2.1, 1.2, and 0.1%, respectively, under different BeamSize compared with the 2-encoder model. Table 5 shows the experimental results of Lewenshtein distance and ROUGE-L metrics, both of which are set with BeamSize 1. Compared with 1-encoder/2-encoders, crBERT1/crBERT2 improves the Lewenshtein distance metric by 7.1 and 3.5%, respectively. The ROUGE-L metric increased by 2.2 and 0.9%, respectively. According to the experimental results, CodeBERT can be used to learn the deeper semantic features of sequences and integrate the structural features of program diagrams to further improve the effectiveness of the two automated code review tasks in this study.

This study designs another set of comparative experiments, which can answer two questions: First, can CodeBERT architecture be used to improve the effectiveness of the two tasks? The second is to convert the code into a program dependency graph, and then convert it into sequence through a specific method. Can this method further improve the effectiveness of the model? In the comparative experiment, we select three models: the Seq2Seq model [30] (encoder is the bidirectional LSTM network, the decoder is the LSTM model), the Transformer model [29] (1-encoder/2-encoders model), CodeBERT model [10]. Firstly, the experiment of Task 1 and Task 2 is completed by using these three models, and then two tasks are completed by integrating the program dependency graph sequence features on these three models, i.e., seq2seq, seq2seq+graph sequence, transfomer, transformer+graph sequence, CodeBERT, CodeBERT+graph sequence. Its architecture is similar to that in Figure 6 and Figure 7, with the BLEU-4 value of BeamSize 1 as the evaluation metric.

Table 6 shows the experimental results of the comparison of six groups of models. On the whole, the crBERT model proposed in this paper is significantly better than the Seq2Seq model and Transformer model. For the first problem, using the pretraining model CodeBERT can significantly improve the model’s performance. The CodeBERT-based crBERT model is 7.7 and 2.6% points higher on task 1 and task 2, respectively, than the Transformer-based model. This is because although the CodeBERT model and Transformer model have similar structures, CodeBERT can learn prior knowledge of code sequences from massive code data, such as code sequence characteristics, code form collocation, and other information. For the second question, because CodeBERT can use both code language and natural language dual-modal language for pre-training, it plays a role in learning the reviewer’s suggestion in Task 2 and establishing the link between the suggestion information and code information. The crBERT model is 0.2 and 1.4 percentage points higher than the Seq2Seq model in task 1 and task 2, respectively. The Seq2Seq model has little difference in the effect of the crBERT1 model in task 1, this is because CodeBERT uses a location coding mechanism, which can effectively avoid the long-term sequential dependence problem of Seq2Seq model and improve the generalization performance of the model. Therefore, the CodeBERT pretraining model is selected as the architecture model in this study.

It can be found from Table 6 that the effect of code review can be further improved by further integrating graph sequences based on the semantic information of code. After fusing the graph sequence features into the model, the Seq2Seq model increases by 0.7 and 0.2 percentage points, respectively, in task 1 and task 2; the Transformer model increases by 6.1 and 0.9 percentage points, respectively, in task 1 and task 2; the crBERT model increases 1.1 and 1 percentage points, respectively, in task 1 and task 2. This is because: firstly, this paper represents the code as a program dependency graph, which can help to learn structural information about the program such as control dependencies and data dependencies of the code; secondly, the PDG2Seq algorithm transforms the program dependency graph into features corresponding to unique graph sequences, which makes it possible to combine the semantic features of the code while preserving the structural features of the program. The above analysis shows that the PDG2Seq algorithm used in this paper can effectively learn the structural information of the code, enhance the transformation ability of the model, and further improve the effectiveness of the model.

In summary, the CodeBERT model outperforms the Seq2Seq model and Transformer model. This is because, although the CodeBERT model has a similar structure to the Transformer model, CodeBERT is able to learn a priori knowledge about code sequences from large amounts of code data, such as learning the sequential characteristics of the code, the formal collocation of the code, and other information; Additionally, CodeBERT uses a dual code language and natural language. Additionally, CodeBERT is pre-trained in both code language and natural language, which is useful for learning reviewer suggestions and associating this information with code information.

## 6. Conclusions

Code review is an important activity in the software lifecycle. It is a time- and energy-consuming activity. The goal of this study is to improve the efficiency of code review from a deep learning perspective. However, most of the related works on the code review do not sufficiently take into account the structural features of the code. However, the structural features of the code contain rich logical and semantic information, and the effectiveness of the code review algorithm can be further improved.

To address this issue, in this study, we propose an automatic code review method that integrates the structural and semantic features of programs. PDG2Seq transforms the program dependency graph into a unique sequence, which not only captures the structural information of the code, but also enhances the learning of the semantic information of the code. Additionally, this paper uses the pre-training model CodeBERT to learn the deeper features of sequences, and combines the program structure and semantic features to enrich the learning of code features. The experiment shows that the method in this study significantly improves the effect of Task 1 and Task 2 compared to the current advanced method.

In the future, our work in this paper may be extended in the following ways. In terms of code language, the Java-based code review work in this paper can be extended to support more code languages (e.g., Python, C++, etc.). In terms of code review granularity, it can be considered to extend the review granularity from a single method-level to different granularities (e.g., statement-level). Additionally, it might be useful to consider extending the code representation approach proposed in this paper to other areas of automatic software engineering, such as software defect detection, automatic code generation/annotation, etc.

## Figures and Tables

**Table 1:** Depth-first search code sequences.

Edge	I	(c)	(d)
0	(*v*_0_,*v*_1_,A,a,B)	(*v*_0_,*v*_1_,B,a,A)	(*v*_0_,*v*_1_,B,d,A)
1	(*v*_1_,*v*_2_,B,d,A)	(*v*_1_,*v*_2_,A,a,C)	(*v*_1_,*v*_2_,A,c,C)
2	(*v*_2_,*v*_3_,A,c,C)	(*v*_2_,*v*_0_,C,b,B)	(*v*_2_,*v*_0_,C,b,B)
3	(*v*_3_,*v*_0_,C,a,A)	(*v*_2_,*v*_3_,C,c,A)	(*v*_2_,*v*_3_,C,a,A)
4	(*v*_3_,*v*_1_,C,b,B)	(*v*_3_,*v*_0_,A,d,B)	(*v*_3_,*v*_0_,A,d,B)
5	(*v*_2_,*v*_4_,A,b,D)	(*v*_3_,*v*_4_,A,b,D)	(*v*_1_,*v*_4_,A,b,D)

**Table 2:** Graph code sequence.

Edge	The Minimum forward Edge Coding	The Minimum Graph Sequence Coding
0	(*v*_0_,*v*_1_,a,0,b)	(*v*_0_,*v*_1_,I,0,b)
1	(*v*_1_,*v*_2_,b,n,d)	(*v*_1_,*v*_2_,b,n,d)
2	(*v*_2_,*v*_3_,d,0,e)	(*v*_2_,*v*_3_,d,0,e)
3	(*v*_2_,*v*_4_,d,0,f)	(*v*_2_,*v*_4_,d,0,f)
4	(*v*_0_,*v*_5_,a,0,c)	(*v*_4_,*v*_2_,I,i,d)
5		(*v*_4_,*v*_3_,f,i,e)
6		(*v*_0_,*v*_5_,a,0,c)
7		(*v*_5_,*v*_2_,c,0,d)

**Table 3:** BLEU experiment results for task 1.

Model	Beam1	Beam3	Beam5	Beam10
1-encoder	0.692	0.769	0.791	0.814
crBERT1	0.78	0.83	0.846	0.862

**Table 4:** BLEU experiment results for task 5.

Model	Beam1	Beam3	Beam5	Beam10
1-encoder	0.692	0.769	0.791	0.814
crBERT1	0.78	0.83	0.846	0.862

**Table 5:** Leweinshtein distance and rouge-L test results.

	Metric	Levenshtein Distance	ROUGE-L
Model			
1-encoder		0.254	0.905
crBERT1		0.183	0.927
2-encoders		0.202	0.926
crBERT2		0.167	0.935

**Table 6:** Ablation results.

Model	Task1	Task2
seq2seq	0.767	0.775
seq2seq+gs	0.773	0.777
tranformer	0.692	0.763
Transformer+gs	0.753	0.772
codeBERT	0.769	0.789
codeBERT+gs	0.78	0.799

## Data Availability

Not applicable.
